# pH-Responsive Cobalt(II)-Coordinated Assembly Containing Quercetin for Antimicrobial Applications

**DOI:** 10.3390/molecules28145581

**Published:** 2023-07-22

**Authors:** Giuseppina D. G. Santonoceta, Carmelo Sgarlata

**Affiliations:** Department of Chemical Sciences, University of Catania, Viale Andrea Doria 6, 95125 Catania, Italy; giuseppina.santonoceta@phd.unict.it

**Keywords:** quercetin, antimicrobial agents, Co(II) complexes, pH-responsive systems, drug delivery, solution equilibria and thermodynamics, speciation, ITC calorimetry, QCM-D, solid–liquid interface

## Abstract

The development of novel drug delivery systems (DDSs) with promising antibacterial properties is essential for facing the emergency of increasing resistance to antimicrobial agents. The antibacterial features of quercetin and its metal complexes have been broadly investigated. However, several drawbacks affect their activity and effectiveness. In this work, we propose a DDS based on a pH-responsive cobalt(II)-coordinated assembly containing quercetin and polyacrylic acid. This system is suggested to trigger the release of the model drug in a pH-dependent mode by exploiting the localized acidic environment at the bacterial infection sites under anaerobic conditions. The delivery system has been designed by accurately examining the species and the multiple equilibria occurring in solution among the assembly components. The formation of cobalt(II) complexes with quercetin in the absence or presence of the pH-responsive polyacrylic acid was investigated in buffered aqueous solution at pH 7.4 using spectrophotometric (UV-Vis) and calorimetric (ITC) techniques. The determined binding affinities and thermodynamic parameters that resulted are essential for the development of a DDS with improved binding and release capabilities. Furthermore, the affinity of the polymer–cobalt(II) complex toward the model antimicrobial flavonoid was explored at the solid–liquid interface by quartz crystal microbalance (QCM-D) experiments, which provided marked evidence for drug loading and release under pH control.

## 1. Introduction

**Figure 1:**
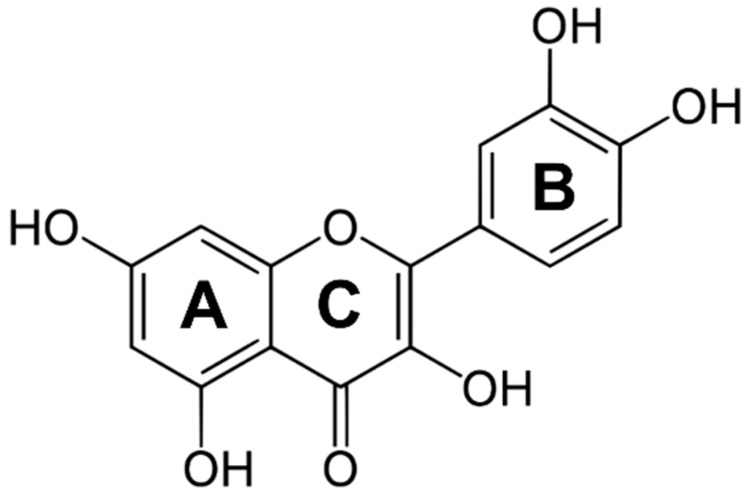
Structure of quercetin.

The misuse and administering of a huge amount of antibiotics led to the sudden rise in antibiotic-resistant bacterial infections, which are nowadays a critical challenge [1,2]. Several strategies, such as the development of new antibiotics or their chemical modification, combinatorial therapy, photothermal agents, antimicrobial peptides, and cationic polymers, have been proposed to face this global issue [3]. In the search for new antimicrobial agents with improved activity and a targeted mode of action, flavonoids, a class of natural compounds, have gained particular interest. Their chemical diversity makes flavonoids versatile molecules exhibiting many biologically and pharmacologically relevant properties, including anti- or pro-oxidant, anti-inflammatory, and anticancer and antimicrobial activity [4,5]. Quercetin (3,5,7,3′,4′-pentahydroxyflavone, Que, Figure 1) is one of the most popular and biologically active compounds among them [6,7,8].

This multi-functional molecule has been proven to have potent antimicrobial activity against a variety of bacterial strains, particularly those affecting the gastrointestinal, respiratory, and urinary tracts [9]. Analogous to other flavonoids [10,11,12], the quercetin antibacterial effect is based on its structural functionalities, as the hydroxyl groups in the flavonoid backbone play a significant role in the interactions with the bacterial cell membrane [13]. Quercetin and its derivatives have been found to be effective agents against several Gram-positive (e.g., methicillin-resistant *S. aureus* (MRSA), methicillin-sensitive *S. aureus* (MSSA), and *Enterococcus*) [14,15] and Gram-negative (e.g., *E. coli*, *K. pneumoniae*, and *P. aeruginosa*) bacteria [16]. However, the difference in the cell membrane composition between the two types of bacteria is responsible for the reduced bactericidal efficacy of the flavonoid towards the Gram-negative compared to the Gram-positive bacteria [17]. Various molecular mechanisms seem to be responsible for its antimicrobial activity [18], such as the ability to disrupt cell membrane integrity [19] and DNA synthesis [20]. Recent studies reported that quercetin effectively decreases biofilm formation, mitochondrial dysfunction, and the production of various virulence factors [21].

The antibacterial features of metal complexes containing flavonoids have been widely investigated [3], and their properties strongly depend on the metal ions, ligands, and bacterial strain. Most of them exhibited an increased antimicrobial activity compared to the free flavonoid (e.g., the Cu(II) complexes of baicalein or naringin as well as the Mg(II) and Ca(II) complexes of morin) [22,23]. Complexes of Hg(II), Mn(II), Co(II), and Cd(II) with quercetin showed remarkable properties against *S. aureus, B. cereus, P. aeruginosa, E. coli*, and *K. pneumoniae* [3]. Although quercetin and its metal complexes have significant antimicrobial activity, their poor solubility in water, limited permeability, and short biological half-life are responsible for their low bioavailability. Many efforts have been made to develop effective delivery systems able to increase the biological activity and availability of the flavonoid in order to produce significant therapeutic effects [24].

Delivery systems actively targeting bacteria have received considerable attention in recent years. Various nanoparticles have been developed, including liposomes, silica, metal, and polymeric nanoparticles [25]. In this context, the development of stimuli-responsive platforms may enable the targeting of the bacteria through recognition of the bacterial microenvironment. The proper design of these systems permits them to respond to internal (e.g., pH, variation in the redox gradient, enzymes) or exogenous (e.g., light, ultrasound) stimuli. Overall, the application of these systems that can release antimicrobials “on-demand” represents a promising strategy to potentially enhance therapeutic efficacy while also reducing drug resistance and side effects [26]. pH-responsive systems have gained a special interest since many bacteria can produce lactic acid and acetic acid during their growth and metabolism, leading to localized acidification at infection sites (pH 4.5–6.5) [27,28].

Natural and synthetic polymeric carriers have been widely employed due to their biocompatibility, degradability, and stability, as well as tunable chemical, interfacial, and mechanical properties, which make them fascinating tools for biological applications [29]. Several investigations have been focused on the controlled release of antibiotics by using pH-responsive agents (such as poly(acrylic acid) (PAA) and poly(methacrylic acid) (PMAA)) [30].

PAA is a synthetically biocompatible hydrophilic polymer and has been one of the most frequently used platforms for the development of pH-responsive drug delivery systems (DDSs). It can retain large amounts of water in its three-dimensional network, and its conformational features (shrinking and expansion of the polymer chain) depend on the pH value. PAA contains carboxylic acid functional groups, which are responsible for its ability to respond to pH changes [31].

As the binding properties of a carrier towards a specific drug and the related thermodynamic signature can reflect its loading and release capability, here we report a strategy based on the study of solution equilibria for the efficient design and development of a delivery system for quercetin (proposed as an antimicrobial model drug) based on a metal ion and poly(acrylic acid) complex.

Due to the presence of carbonyl and hydroxyl groups, Que can coordinate metal ions and form complexes, which should have better antioxidant properties and biological effects [32]. The metal ions can be coordinated by three different domains: the 3′,4′-dihydroxy group on the B ring as well as the 3- or 5-hydroxy and the 4-carbonyl group in the C ring of the Que molecule (Figure 1) [33]. Quercetin’s ability to bind ions of biological interest (iron, copper, zinc) has been examined, and several complex species with different stoichiometries have been proposed [34,35,36]. In this framework, the intriguing biological properties (antibacterial, antiviral, and antioxidant) of cobalt(II) complexes [37,38] inspired our investigation of a cobalt(II)-based assembly containing quercetin and PAA as the carrier able to respond to pH change. The formation of the Que-Co(II)-PAA adduct was examined both in solution by UV-Vis and isothermal titration calorimetry (ITC) and at the solid–liquid interface by quartz-crystal microbalance with dissipation monitoring (QCM-D), which also allowed assessment of the release of the drug under pH stimuli.

Overall, this carefully designed assembly should improve flavonoid bioavailability and enable its pH-controlled release. In addition, the resulting DDS could display antimicrobial properties, as it should be able to interact with the phospholipid components in the cytoplasmic membrane, causing bacterial cell membrane disruption.

## 2. Results and Discussion

The cobalt(II)-based polymeric assembly containing quercetin was developed in aqueous solution at neutral pH by exploiting the ability of the various components to establish multiple non-covalent interactions. An accurate design of the final DDS implies the investigation of the multiple equilibria occurring in solution among the constituents of the system. Consequently, the binding affinities and thermodynamic parameters for the complexation of Co(II) with Que and poly(acrylic acid), as well as for the formation of the Que-Co(II)-PAA assembly, were determined. Previous studies have shown that PAA and quercetin are not able to interact due to the electrostatic repulsion that occurs between the negatively charged moieties of the two molecules under our experimental conditions [39].

### 2.1. Quercetin–Cobalt(II) Complex

**Figure 2:**
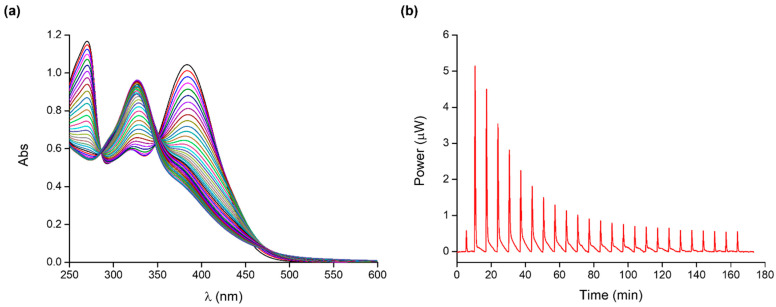
Typical (**a**) UV-Vis titration of Co(II) (0.60 mM) into Que (0.06 mM) and (**b**) ITC titration of Co(II) (0.55 mM) into Que (0.12 mM) at 25 °C and pH 7.4; the integrated heat data are shown in Figure S1.

An essential step for the development of cobalt(II)-coordinated assembly is the study of the complex species that quercetin can form with the metal ion. For example, Bukhari et al. [40] proposed a 2:1 (metal: ligand) complex in methanol, while Kalinowska et al. [41] reported a 1:2 (metal: ligand) complex in Tris-HCl at pH 7.4. As the stoichiometry of quercetin complex species strongly depends on the experimental conditions, we decided to study the formation of Co(II)-Que complexes in buffered aqueous solution at pH 7.4 (MOPS 10 mM). MOPS, an N-substituted aminosulfonic acid with a morpholine ring, was chosen as the buffering agent, as it does not bind metals and is commonly used for the study of metal complexes in solution. Species, binding affinities, and thermodynamic parameters of quercetin–cobalt(II) complexes have been determined using UV-Vis and ITC calorimetric measurements; typical titrations are shown in Figure 2.

Quercetin absorption bands at 379 nm (band I) and 269 nm (band II) are ascribable to the π→π* transitions within the aromatic three-ring system of the ligand molecule. Specifically, band I is due to the absorption of the cinnamoyl system (ring B), while band II to that of the benzoyl moiety (ring A). Both bands show a decrease in absorbance upon the addition of the metal ion to the ligand solution. Moreover, the extension of the conjugated system as a consequence of the metal–ligand complexation process determines a concomitant slight red shift of band I. Comparison with the literature prompted us to suppose that, at our experimental conditions, the 3-hydroxyl and 4-oxo groups of quercetin are the sites involved in the complexation process [42,43].

Multi-wavelength analysis of the spectroscopic data in the 240–480 nm range allowed for the accurate determination of the cobalt(II) complex species and their conditional stability constants at pH 7.4. Different species and their combinations were tested with the program, but the data analysis consistently converged to the results shown in Table 1.

To our knowledge, a detailed study on the quercetin binding ability toward cobalt(II) in water at pH 7.4 has not yet been reported. In agreement with the stoichiometry reported for several flavonoid complexes, quercetin forms quite stable ML and ML_2_ species with cobalt(II). Due to the very low solubility in aqueous solution, in many cases, the complex stability was determined in organic solvent or water/organic solvent mixtures, and consequently, this makes the data comparison rather difficult. Indeed, the polarity of the solvent and its capability of forming hydrogen bonds significantly affect the metal ion–ligand interactions. The stability constant value found for the ML species at pH 7.4 is quite similar to those reported by Malesev et al. [44] for the complexes formed by cobalt(II) with quercetin and rutin at pH 5.

The formation of quercetin–cobalt(II) complexes was also examined by calorimetric titrations to obtain a complete thermodynamic characterization of the investigated system. The binding parameters are shown in Table 1. The ITC measurements confirm that quercetin forms ML and ML_2_ complex species with cobalt(II). In both cases, the complexation process is enthalpically favoured; the formation of the ML species is driven by entropy due to metal and ligand desolvation upon complexation. The binding of a second ligand molecule (ML_2_ species) is driven by enthalpy with an unfavourable entropic contribution ascribable to the loss of degrees of freedom of the system as a result of the complexation process.

### 2.2. Poly(acrylic acid)–Cobalt(II) Complex

The binding properties of water-soluble chelating polymers and the ability of their functional groups to bind metal ions have been extensively studied. The stability and the features of these complexes depend on several factors (nature of the polymer backbone, distance of the pendant binding sites from the backbone, nature of the metal ions, pH, ionic strength) [45], and they can be stabilized by electrostatic, donor-acceptor, and hydrophobic interactions [46].

The cobalt(II)–polymer complex species formation was investigated by ITC calorimetric titrations (Figures S2 and S3), and the resulting species, binding constants, and thermodynamic parameters are in Table 2.

Poly(acrylic acid) forms M(PAA) and M(PAA)_2_ complex species, which show similar binding affinities. Based on the literature reports on the possible binding modes with similar ligands, we can suppose that “*two coordinations of the metal ion are satisfied by the two carboxyl anions, and the residual two coordinations are satisfied by the oxygen of two carboxyl groups in the polymer*” [47,48,49].

The formation of both poly(acrylic acid)–cobalt(II) complexes is entropically favoured and driven due to the orientation disorder of hydration water molecules upon complexation and conformational changes of the polymer backbone. The formation of M(PAA) complex species is enthalpically unfavoured, while a slightly favourable enthalpic contribution is observed for M(PAA)_2_ (the binding of a second carboxyl unit to the metal ion compensates for the cost in energy needed for desolvation).

### 2.3. Quercetin–Cobalt(II)–Polymer Assembly

As cobalt(II) can form complexes with both quercetin and polyacrylic acid in solution, we decided to rationally design and form the expected DDS based on Que, Co(II), and PAA by taking advantage of the interactions occurring among these components as well as the quantitative information on the species and binding affinities experimentally determined at pH 7.4.

The metal–polymer complex was first prepared in solution by mixing cobalt(II) and poly(acrylic acid) in a proper molar ratio. Thus, once the concentration of the polymer was fixed (C_PAA_ = 9.00 mM), we prepared solutions at different metal/polymer ratios, trying to increase the number of possible binding sites (i.e., metal ions that could act as anchoring/bridging points) between the polymer and the flavonoid that otherwise could not interact. Cobalt(II)–polymer solutions prepared at 1:1 or 1:2 molar ratios resulted in the formation of a precipitate upon the addition of the metal ion to the polymer solution. A lower concentration of the metal ion would avoid the precipitation of the metal–polymer complex; therefore, we mixed Co(II) and PAA at a 1:3 molar ratio to have the largest possible number of binding sites while preventing precipitation events. The combination of carefully selected PAA concentration and metal/polymer ratio permitted having the optimal number of potential binding sites for quercetin. Knowledge of the polymer’s affinity toward the cobalt(II) ion and a simple species distribution calculation (Figure S4) indicated that, under our conditions, M(PAA)_2_ is the main species present in solution. The diagram in Figure S2 suggests that about 60% of the polymer is complexed with the metal ion, while the remaining 40% is free. To reduce the amount of free polymer, a higher metal–polymer ratio would be needed, with consequent precipitation danger; however, the non-complexed polymer in solution is not an issue, as it is not able to bind quercetin. Moreover, PAA is reported to be a non-toxic, biocompatible, and biodegradable polymer, and its apparently high concentration should not have significant side effects in view of possible biological applications (which usually require solutions obtained by diluting the assembly stock solution).

**Figure 3:**
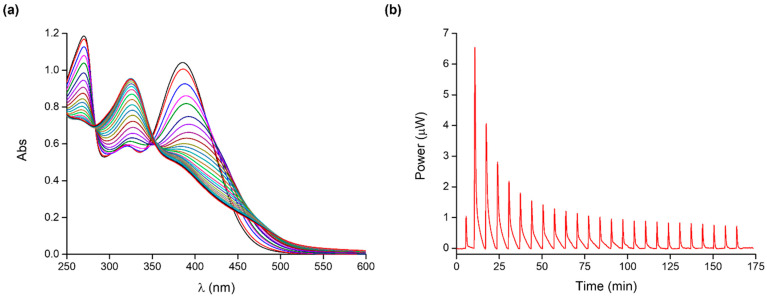
Typical (**a**) UV-Vis titration of cobalt(II)–polymer complex (Co(II):PAA 1:3; C_PAA_ = 9.00 mM) into Que (0.06 mM) and (**b**) ITC titration of cobalt(II)–polymer complex (Co(II):PAA 1:3; C_PAA_ = 9.00 mM) into Que (0.15 mM) at 25 °C and pH 7.4; the integrated heat data are shown in Figure S5.

Both UV-Vis and ITC measurements were carried out to determine species, stability constants, and energetics of the assembly formation process in solution; typical UV-Vis and ITC titrations are shown in Figure 3.

The intensity of the quercetin bands gradually decreases upon the stepwise addition of the cobalt(II)–polymer complex to the ligand solution (Figure 3a). A simultaneous red-shift of band I is observed, which is more evident than that for the UV-Vis titration of the free cobalt(II) into the flavonoid. Also, no changes in the position of band II are observed. Two isosbestic points at 285 and 352 nm could be detected, indicating that quercetin could form complex species with the cobalt(II)–poly(acrylic acid) system. Since quercetin cannot bind the free polyacrylic acid, the metal ion proved to be essential for the formation of the final assembly, as it acts as a bridging unit between Que and PAA.

For data analysis, all the possible equilibria involving the system components and their binding parameters were considered (i.e., the metal–ligand and metal–polymer complex species described in Table 1 and Table 2) and included in the overall model. The interaction of the cobalt(II)–polymer complex with quercetin led to the formation of the LM(PAA)_2_ species. The determined binding affinity and parameters are shown in Table 3.

The QueCo(II)(PAA)_2_ complex formation is an enthalpically and entropically favoured process; enthalpy and entropy changes contribute almost comparably to the Gibbs free energy of the reaction. The favourable entropic term is due to the desolvation of both quercetin and the polymer–metal complex; the enthalpy gain, which prevails on the energy cost for desolvation, is attributable to the favourable attractive forces between Que and the metal ion. This enthalpic term, which is comparable to that determined for the formation of the Co(II)-Que species, supports the idea that the coordination features of the metal ion in the final three-component assembly are satisfied by the quercetin functional groups and two carboxyl groups of the polymer chain.

Since the system has been developed to increase the bioavailability of quercetin and to enhance its antimicrobial activities, accurate knowledge of the complex species that could be actually responsible for these biorelevant properties is of paramount importance. These studies on solution equilibria and speciation are hence indispensable for optimizing the design of the drug delivery systems and ensuring their effectiveness towards the target cells.

### 2.4. Assembly Formation at the Interface and Drug Release at Controlled pH

The formation of the metal-coordinated assembly was also studied at the solid–liquid interface by quartz-crystal microbalance with dissipation monitoring (QCM-D). This is a real-time, surface-sensitive technique for analyzing surface-interaction phenomena, film formation, and layer properties. The high sensitivity of the technique enables monitoring the adsorption and desorption of small molecular systems, such as drugs, without the use of probes [50,51,52]. The molecule–surface interactions are detected as changes in mass (mass uptake or loss) as molecules adsorb or desorb on/from the surface. Drug uptake and release are detected in real time by monitoring changes in the frequency oscillation and energy dissipation of a gold-coated quartz sensor upon absorption/desorption. Both kinetic and thermodynamic parameters related to complexation processes occurring at the interface may be determined [53].

**Figure 4:**
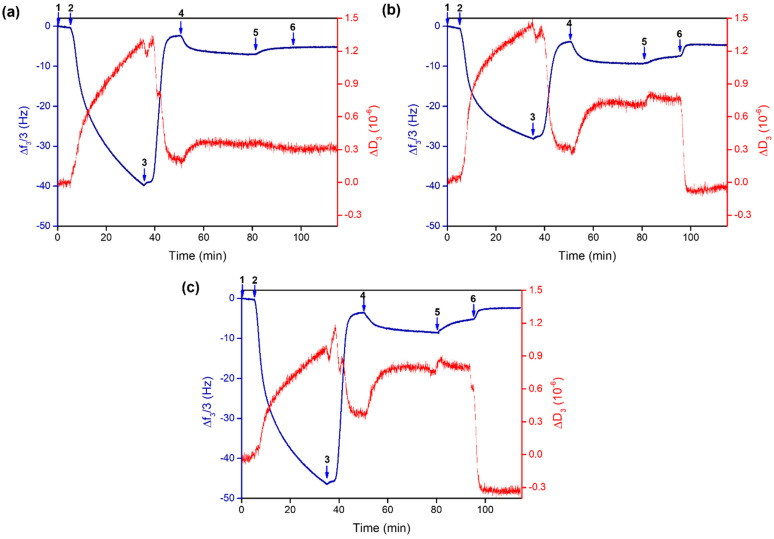
Normalized frequency ΔF_n_/n (blue) and dissipation ΔD_n_ (red) change (for simplicity, only data referring to the third overtone are shown, n = 3) for the adsorption of the cobalt(II)–polymer layer onto a gold-coated quartz crystal and subsequent loading and release of quercetin at 25 °C. (1) Baseline: 10 mM MOPS at pH 7.4; (2) adsorption of the cobalt(II)–polymer system (M:PAA 1:3, C_PAA_= 9.00 mM) in 10 mM MOPS at pH 7.4; (3) rinsing with 10 mM MOPS at pH 7.4; (4) uptake of quercetin from its solution (C_Que_ = 0.15 mM) in 10 mM MOPS at pH 7.4; (5) rinsing with 10 mM MOPS at pH 7.4; (6) rinsing with 10 mM MOPS at (**a**) pH 7.4, (**b**) pH 5.4, and (**c**) pH 4.5.

Figure 4 shows the observed changes in frequency (Δ*F*) and dissipation (Δ*D*) in a typical QCM-D experiment; for simplicity, only data relating to the third overtone (Δf_3_/3 and ΔD_3_) are reported.

A rapid decrease in frequency is observed after step 2 (see Figure 4) due to the adsorption of the metal–polymer system to the sensor surface at pH 7.4; this also results in an increase in energy dissipation, owing to the less-rigid structure of the adsorbed layer of the cobalt(II)–PAA complex onto the gold sensor [54]. During the subsequent rinsing step, a significant decrease in the dissipation is observed with time, likely due to the rearrangement of the film, resulting in a more compact, rigid layer. It should be pointed out that, in both cases, the dissipation value is fairly small, with the |ΔD_n_/(Δf_n_/n)| ratio being much smaller than 4 × 10^−7^ Hz^−1^ [55]; consequently, we can consider the adsorbed film as basically rigid. Thus, the Sauerbrey relationship may be used to convert the change in frequency to the adsorbed mass.

The adsorption of quercetin onto the polymeric layer (step 4) caused a decrease in frequency, and we can assume that the flavonoid could be trapped in the adsorbed metal–polymer layer thanks to hydrophobic interactions and/or by metal-ion coordination. The frequency increase observed after the rinse in step 5 corresponds to the removal of the non-adsorbed drug.

Interestingly, differences were observed after flowing either neutral or acidic solutions (step 6), which were chosen to investigate the ability of the drug to be released from the adsorbed metal-coordinated assembly at different pH values. The flowing of a neutral solution (pH 7.4, Figure 4a) does not cause major frequency variations, whereas when an acidic solution is flowed (pH 5.4 and 4.5, Figure 4b,c), a decrease in the frequency—up to values similar to those obtained after the removal of the non-adsorbed cobalt(II)–polymer complex layer (step 3)—is observed. This evidence may be explained by assuming a pH-induced breakdown of the coordination bonds between quercetin and cobalt(II) ions (which act as the anchoring sites to the polymer chain) and the consequent release of the flavonoid from the assembly. A complementary pH-dependent swelling mechanism that involves the protonation of the free carboxyl groups of the polymer might also be invoked [30]. As pH decreases (and approaches the pK_a_ value of 4.5), the equilibrium between –COO^−^ and –COOH groups shifts towards COOH; therefore, the overall charge of PAA decreases with a consequent rearrangement of the polymer chains, which further contributes to the release of the drug that was supposed to be physically trapped into the polymeric layer.

**Figure 5:**
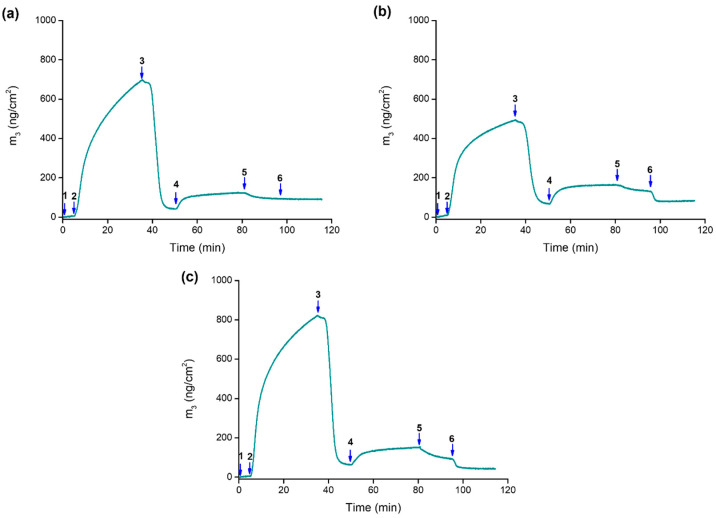
Mass changes (Δm) for the cobalt(II)-coordinated assembly adsorption onto a gold-coated quartz crystal and subsequent loading and release of quercetin at 25 °C at (**a**) pH 7.4, (**b**) pH 5.4, and (**c**) pH 4.5. The conditions for each flowing step are as described in the caption of Figure 4.

The plot of the adsorbed mass (ng/cm^2^), calculated using the Sauerbrey equation, is shown in Figure 5.

The drug loading into the cobalt(II)–polymer layer as well as its subsequent release are summarized in Table 4. The normalized release profiles at different pH values are shown in Figure S6. It is noteworthy that a very low amount of quercetin is released under neutral conditions (ca. 6%); conversely, under acidic conditions, more than 60% of the adsorbed quercetin is released by the assembly. This evidence proves that the developed delivery system can respond to changes in the pH value, thus allowing a controlled release of the loaded drug. This is significant to guarantee an adequate concentration of the drug at the infection site, increasing the antimicrobial activity and minimizing the development of bacterial resistance [56].

The time dependency of the frequency (and thus of the mass) changes also allowed us to determine the kinetic rate constants (*k_on_* and *k_off_*) as well as the association constant (K_ass_) at the solid–liquid interface [57,58]. As widely reported in the literature, to quantitatively determine these parameters, both the adsorption and desorption processes have to follow the Langmuir model and fulfill its relevant assumptions [59].

The binding process may be described by the following reaction:

*k_on_* L + M(PAA)_2_ ⇄ LM(PAA)_2_

*k_off_* (1)

According to the Langmuir model, the adsorption process of the ligand can be described by the function:

*θ*(*t*) = *θ_eq_* · (1 − *e^−(kon·C+koff)·t^*) (2)

in which:

- *θ(t)* is the time-dependent surface coverage,
- *θ_eq_* is the concentration-dependent equilibrium surface coverage,
- *k_on_* and *k_off_* are the kinetic rate constants for the binding and unbinding process,
- *C* is the concentration of the adsorptive ligand;

whilst the desorption process is described by the following equation:

*θ*(*t*) = *θ_eq_* · *e^−koff·t^* (3)

**Figure 6:**
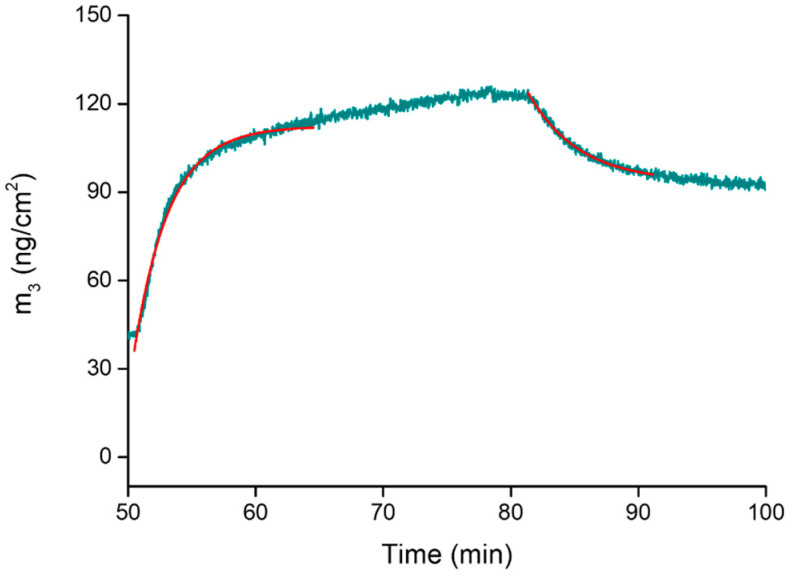
Curve fitting of the adsorption and desorption profiles. The curve is a magnification of steps 4 and 5 of the mass changes plot in Figure 5; the experimental curve is in dark cyan, while the calculated ones are in red.

The analysis of the kinetics of adsorption and desorption through Equations (2) and (3) (Figure 6) allowed us to determine the rate constants *k_on_* and *k_off_* as well as to calculate the association constant K*_ass_* = *k_on_*/*k_off_*.

The kinetic rate constants are *k_on_* = 634 ± 87 M^−1^ min^−1^ and *k_off_* = 0.21 ± 0.04 min^−1^. Using these values, a K*_ass_* of 3019 ± 87 M^−1^ was calculated at the solid–liquid interface (log K*_ass_* = 3.47 (1)), which is in good agreement with that determined in solution by UV-Vis/ITC experiments.

## 3. Materials and Methods

### 3.1. Chemicals

Quercetin (Que) (purity ≥ 95%, HPLC), cobalt(II) perchlorate hexahydrate (purity ≥ 98%), 4-morpholinepropanesulfonic acid (MOPS) (purity ≥ 99.5%, titration), dimethyl sulfoxide for spectroscopy (DMSO) (purity ≥ 99.8%), and poly(acrylic acid sodium salt) (PAA) (average Mw ~5100, GPC, purity ≥ 99%) were purchased from Sigma-Aldrich and used without purification. Stock solutions of Que were prepared in pure DMSO and then properly diluted in a buffered aqueous solution (pH 7.4, MOPS 10 mM) to have a final organic solvent content of only 5% v/v. Buffer solutions were prepared by weighing proper amounts of MOPS and titrating with a sodium hydroxide solution to the desired pH. All Que solutions were always freshly prepared and stored in the dark to avoid possible light-induced alterations. The cobalt(II) stock solution was prepared by dissolving the corresponding perchlorate salt in water and titrating the resulting solution with standard EDTA, using orange xylenol as an indicator [60]. Poly(acrylic acid) stock solutions were prepared at pH 7.4 in MOPS 10 mM; the concentration of the polymer was expressed per monomer unit of acrylic acid. High-purity water (Millipore, Milli-Q Element A 10 ultrapure water) and A-grade glassware were employed throughout.

### 3.2. UV-Vis Titrations

UV-Vis titrations were carried out at 25 °C in buffered aqueous solution (pH 7.4, MOPS 10 mM) with a V-770 UV-Vis/NIR spectrophotometer (Jasco Europe, Italy) in a 1 cm path-length quartz cell. Increasing amounts of the proper titrant solutions were added with a precision pipette (Gilson) into the cell containing 2 mL of the sample solution. All solutions were prepared to have the same % (*v*/*v*) amount of DMSO. The reaction mixture in the cell was stirred during the titrations. Injection time intervals were chosen to ensure equilibrium conditions before each subsequent addition.

*Quercetin–cobalt(II) complex:* UV-Vis titrations were conducted by adding a solution of Co(II) (0.4–1.0 mM) into a Que solution (0.05–0.06 mM).

*Quercetin–CoPAA_2_ assembly:* The interactions occurring between the cobalt(II)-polyacrylic acid complex and Que were investigated by titrating the flavonoid (0.05–0.06 mM) in the cell with a metal–polymer solution prepared by properly mixing Co(II) and PAA at a 1:3 molar ratio (C_PAA_ = 9.00 mM).

About 30 spectra were recorded for each titration. The analysis of the spectral data was performed using Hyperquad software (version 3.1.60) [61], which is specifically designed to determine equilibrium constant and molar absorptivity values. The program allows for a multi-wavelength treatment of the spectral data through a non-linear least-squares minimization procedure and for the simultaneous refinement of data obtained from different titrations.

### 3.3. Isothermal Titration Calorimetry (ITC) Measurements

Calorimetric titrations were performed at 25 °C in buffered aqueous solution (pH 7.4, MOPS 10 mM) with a nano-isothermal titration calorimeter Nano-ITC (TA Instruments, New Castle, DE, USA). Titrant solutions contained in a 250 μL syringe were injected into sample solutions placed in the cell with an active volume of 0.988 mL. Injection time intervals were chosen to guarantee equilibrium conditions before each subsequent addition. The reaction mixture in the sample cell was stirred at 250 rpm during the titration. The reference cell was always filled with ultrapure water. All solutions were stirred and degassed under vacuum for about 15 min before each run. Measurements were run in the overfilled mode. At least three titrations were carried out per system. 

*Quercetin–cobalt(II) complex:* ITC titrations were carried out by titrating a solution of Que (0.08–0.12 mM) with Co(II) (0.50–0.60 mM).

*Poly(acrylic acid)–cobalt(II) complex:* Cobalt–polymer complex formation was investigated by titrating a solution of Co(II) (3.00–3.50 mM) into PAA solutions (0.25–0.50 mM).

*Quercetin–CoPAA_2_ assembly:* Calorimetric titrations were conducted by titrating a metal–polymer solution at a Co(II):PAA 1:3 molar ratio (C_PAA_ = 9.00 mM) into a Que solution (0.12–0.15 mM).

Heats of dilution were determined in separate “blank” experiments by titrating the proper titrant solution into the buffer solution (pH 7.4, MOPS 10 mM). In all experiments, the % (*v*/*v*) of DMSO in the syringe and the cell was the same to avoid heat effects caused by solvent dilution. The power curve was integrated by NanoAnalyze (TA Instruments, New Castle, DE, USA) to obtain the gross heat of the reaction. The calorimeter was calibrated chemically by a test HCl/TRIS reaction according to the procedure described [62]. The instrument was also checked by electrical calibration. The net heats of reaction, obtained by subtracting the heat evolved/absorbed in the blank experiments, were analyzed using HypCal software (version 2.0.34) [63], which enables the determination of both equilibrium constants and enthalpies of complex formation [64,65] through a non-linear least-squares minimization of the calorimetric data obtained from different titrations.

### 3.4. Quartz Crystal Microbalance with Dissipation Monitoring (QCM-D) Experiments

QCM-D is an interface-specific technique based on changes in frequency and energy dissipation of a gold-coated quartz crystal upon material adsorption, which is recorded at multiple overtones of the crystal fundamental resonance frequency. This parameter is sensitive to the mass of the adsorbed film and to its viscoelastic properties [66,67]. At certain conditions, the use of the microbalance is based on the linear relationship between changes in the resonator mass and in the resonance frequency according to the Sauerbrey equation

(4) $$\Delta m=-C*\frac{\Delta f}{n}$$

where Δ*m* is the mass area change (in ng cm^−2^), *C* is the mass sensitivity of the sensor (17.7 ng cm^−2^ Hz^−1^ at the oscillation frequency of 5 MHz), and *n* is the overtone number [55].

QCM-D measurements were performed using a QSense E1 instrument (Biolin Scientific, Gothenburg, Sweden) in a flow-through cell equipped with a precise temperature controller, which contained the sensor crystal, and was connected to a peristaltic pump that forced the solution through the chamber. The sensor crystals were gold-coated AT-cut quartz with gold-plated polished electrodes (QSX 301, Biolin Scientific, Gothenburg, Sweden). All the instrument modules were connected to a computer that monitored the different signals (resonance, oscillation frequency, and temperature). All measurements were carried out at 25 °C and a flow rate of 50 μL/min. Before each experiment, the crystals were thoroughly cleaned according to the QSense cleaning protocol (sequential treatment with ozone for 10 min, a mixture of Milli-Q water/NH_4_OH/H_2_O_2_ = 5:1:1 at 75 °C for 5 min, rinsing with Milli-Q water, drying with a nitrogen flux, and a final ozone treatment for 10 min).

Each measurement started recording the baseline while flowing a MOPS 10 mM, pH 7.4 solution. The polymer–metal complex, prepared by properly mixing cobalt(II) and PAA at 1:3 molar ratio (C_PAA_ = 9.00 mM), was then adsorbed on the sensor crystal surface; a washing step using the same buffer solution was later performed to remove any loosely attached material. Afterwards, a 0.15 mM quercetin solution (in MOPS 10 mM, pH 7.4) was flowed through the adsorbed film until the frequency change reached equilibrium. A washing step was then required to remove the non-adsorbed drug. Finally, a solution of MOPS 10 mM at pH 7.4, 5.4, or 4.5 was flowed until constant frequency changes were recorded to assess the pH-responsiveness of the adsorbed systems. The obtained QCM-D data were analyzed using the integrated Qtools software (version 3.1.27.609) (Biolin Scientific, Gothenburg, Sweden).

## 4. Conclusions

The study of multiple equilibria occurring in aqueous solution at pH 7.4 among quercetin, cobalt(II), and polyacrylic acid is proposed for the design and development of a metal-based assembly containing this flavonoid as a model antimicrobial drug. Combined UV-Vis and ITC experiments enabled the accurate determination of the binding parameters for the species formation in solution. Quercetin forms ML and ML_2_ complex species with cobalt(II) by enthalpically favoured processes. The carboxyl groups of the polyacrylic acid chains allow for the complexation of cobalt(II) ions forming M(PAA) and M(PAA)_2_ species; the complexation process is entropically favoured and driven mostly due to desolvation and conformational changes of the polymer chains. Cobalt(II) ions work as anchoring/bridging sites for the efficient binding of quercetin with the polymer to form the resulting metal-coordinated assembly. Through the analysis of the overall equilibria involved, we have demonstrated that a QueCo(II)(PAA)_2_ complex forms in solution and the process is both enthalpically and entropically favoured.

The affinity of the flavonoid toward the metal–polymer complex was also confirmed at the solid–liquid interface. The determination of the amount of our model antimicrobial molecule loaded into the polymeric layer and its subsequent release under different pH conditions proves that the developed delivery system permits a controlled release of the loaded drug. Overall, this work provides a versatile strategy for the design of a smart antimicrobial system exhibiting a pH-controlled delivery of the antibacterial agent with great potential for the treatment of infection resistance. Future studies will focus on the in vitro antimicrobial activity of the developed metal-coordinated assembly. Moreover, the fate of the Co(II)–polymer species after releasing the drug at the infection site will also be evaluated to have a complete picture of the antimicrobial potential of each component of the proposed system. Indeed, in line with the antibacterial properties reported for cobalt(II) complexes and cobalt-based nanoparticles, the Co(II)–polymer complex might exhibit some favorable synergetic effect, increasing the overall efficacy of quercetin.

## Figures and Tables

**Table 1:** Conditional stability constant values and thermodynamic parameters ^a^ for the quercetin (L)–cobalt(II) (M) complex formation at 25 °C and pH 7.4.

Species	Log *K* UV-Vis	Log *K* ITC	Δ*H*^0^ (kJ mol^−1^)	Δ*S*^0^ (J K^−1^ mol^−1^)
ML	5.66 (2)	5.7 (1)	−11.45 (2)	71 (3)
ML_2_	4.52 (8)	3.3 (2)	−24.33 (4)	−19 (3)

^a^ σ in parenthesis.

**Table 2:** Conditional stability constant values and thermodynamic parameters ^a^ for polyacrylic acid (PAA)–cobalt(II) (M) complex formation at 25 °C and pH 7.4.

Species	Log *K*	Δ*H*^0^ (kJ mol^−1^)	Δ*S*^0^ (J K^−1^ mol^−1^)
M(PAA)	4.3 (2)	7.39 (6)	108 (5)
M(PAA)_2_	3.8 (3)	−1.23 (8)	68 (2)

^a^ σ in parenthesis.

**Table 3:** Conditional stability constant values and thermodynamic parameters ^a^ for the formation of the cobalt(II)-coordinated assembly containing quercetin at 25 °C and pH 7.4.

Species ^b^	Log *K* UV-Vis	Log *K* ITC	Δ*H*^0^ (kJ mol^−1^)	Δ*S*^0^ (J K^−1^ mol^−1^)
LM(PAA)_2_	3.75 (1)	4.5 (2)	−14.17 (6)	38 (5)

^a^ σ in parenthesis; ^b^ refers to the equilibrium qL + M_p_PAA_r_ ⇄ L_q_M_p_PAA_r_.

**Table 4:** Adsorbed mass of quercetin onto the cobalt(II)–polymer layer and % of quercetin released at different pH values at 25 °C.

	Que Loaded	% Que Released		
		pH 7.4	pH 5.4	pH 4.5
Adsorbed mass (ng cm^−2^)	50 ± 12	6%	62%	64%

## Data Availability

Data are available from the corresponding author upon reasonable request.

## Supplementary Materials

The following supporting information can be downloaded at: https://www.mdpi.com/article/10.3390/molecules28145581/s1, Figure S1: Integrated heat data for the cobalt(II) into quercetin ITC titration in Figure 2b; Figure S2: ITC titration of cobalt(II) (3.50 mM) into polyacrylic acid (0.50 mM) at 25 °C and pH 7.4.; Figure S3: Integrated heat data for the cobalt(II) into PAA ITC titration in Figure S2; Figure S4: Distribution diagram for the cobalt(II)–polymer complex species (Co(II):PAA 1:3, C_PAA_ = 9.00 mM) computed by using the data in Table 2; Figure S5: Integrated heat data for the cobalt(II)-PAA complex into quercetin ITC titration in Figure 3b; Figure S6: Release profiles of quercetin from the metal–polymer layer at different pH values. The curves of the (%) amount of Que bound are obtained by normalizing the mass absorption values (i.e., the mass change curves of step 6 in Figure 5) by the maximum amount of Que bound as a function of time.
