# Supplementary material for: pH-Responsive Cobalt(II)-Coordinated Assembly Containing Quercetin for Antimicrobial Applications
Source: Molecules. 2023 Jul 22;28(14):5581. doi: 10.3390/molecules28145581 (PMC10386366; doi:10.3390/molecules28145581)
Supplement: Supplementary file 1 [file molecules-28-05581-s001.zip › molecules-2502544-supplementary.pdf]

pH-Responsive Cobalt(II)-Coordinated Assembly Containing Quercetin for Antimicrobial Applications

Giuseppina D. G. Santonoceta and Carmelo Sgarlata*

Department of Chemical Sciences, University of Catania, Viale Andrea Doria 6, 95125 Catania, Italy;
giuseppina.santonoceta@phd.unict.it; sgarlata@unict.it
* Correspondence: sgarlata@unict.it

Supplementary Material

Table of contents

Figure S1.	Integrated heat data for the cobalt(II) into quercetin ITC titration	**S2**
Figure S2.	Typical ITC titration curve of cobalt(II) into PAA	**S2**
Figure S3.	Integrated heat data for the cobalt(II) into PAA ITC titration	**S3**
Figure S4	Distribution diagram for cobalt(II)-polymer complex species	**S3**
Figure S5	Integrated heat data for the cobalt(II)-PAA complex into quercetin ITC titration	**S4**
Figure S6	Release profiles of quercetin from the metal-polymer layer at different pH values	**S4**

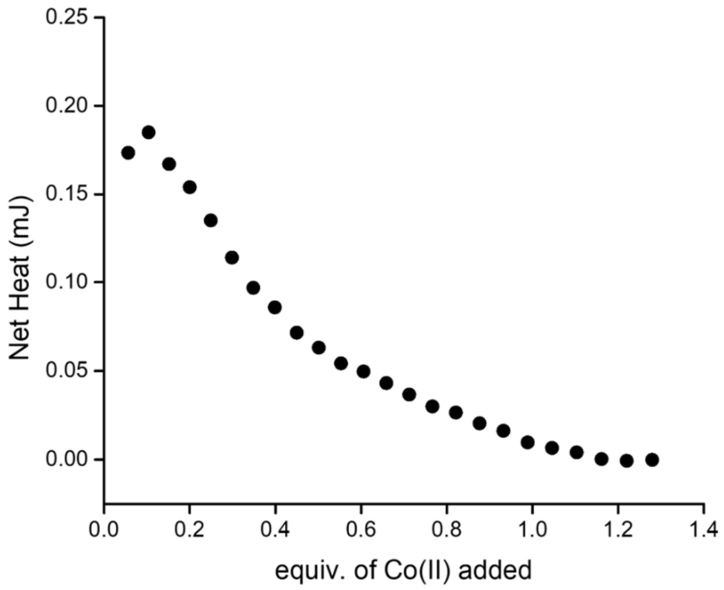

Figure S1. Integrated heat data for the cobalt(II) into quercetin ITC titration in Figure 2b.

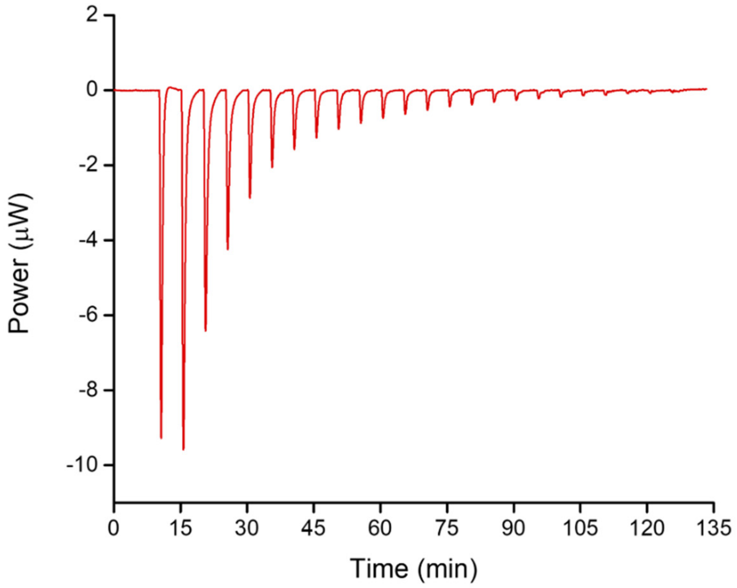

Figure S2. ITC titration of cobalt(II) (3.50 mM) into polyacrylic acid (0.50 mM) at 25 °C and pH 7.4.

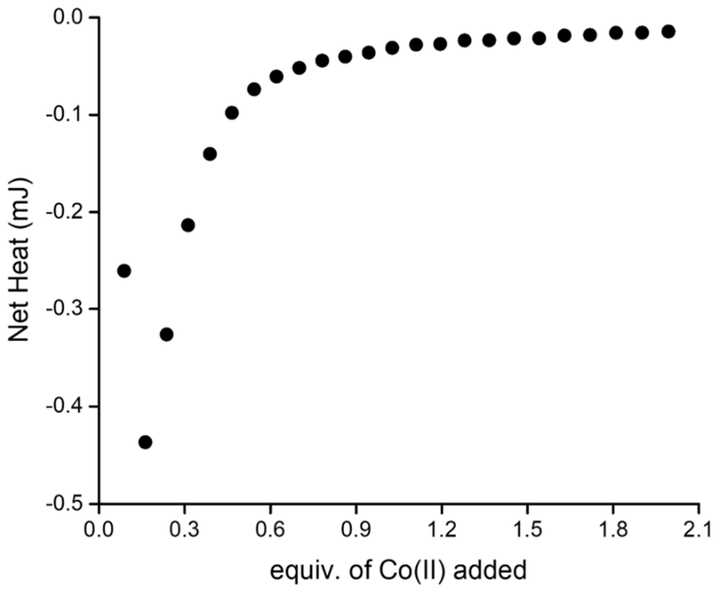

Figure S3. Integrated heat data for the cobalt(II) into PAA ITC titration in Figure S2.

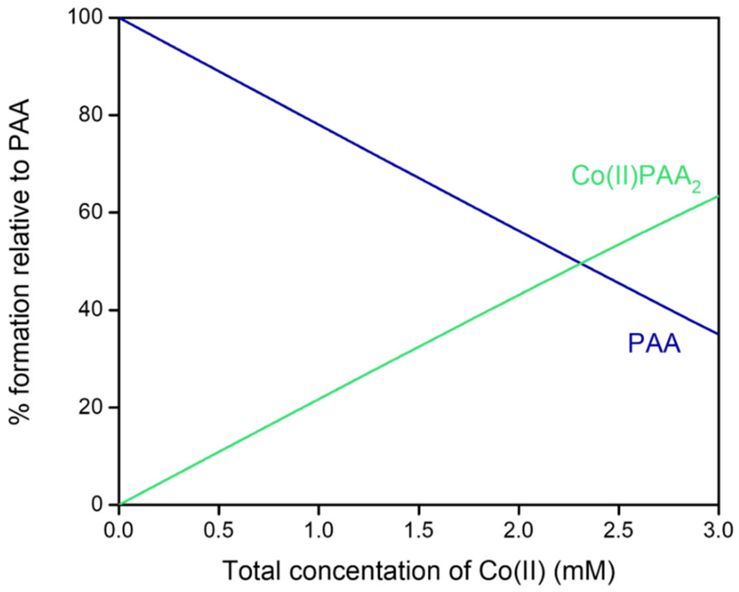

Figure S4. Distribution diagram for the cobalt(II)-polymer complex species (Co(II):PAA 1:3, C_{PAA} = 9.00 mM) computed by using the data in Table 2.

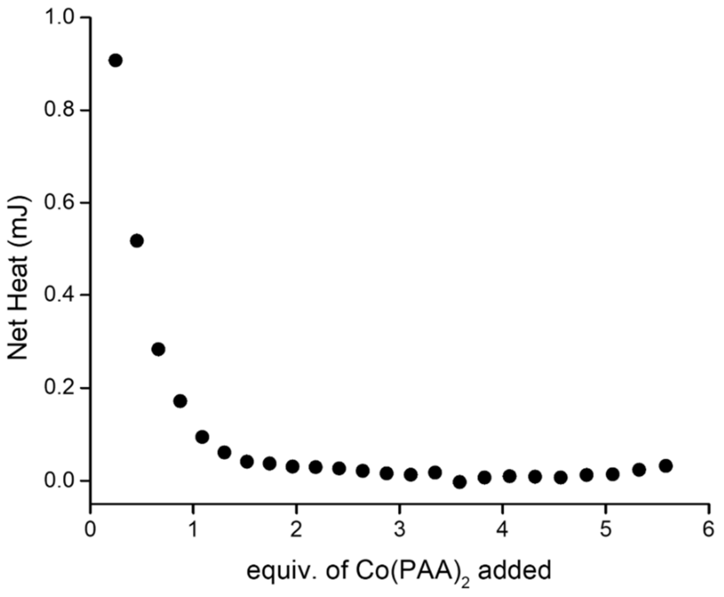

Figure S5. Integrated heat data for the cobalt(II)-PAA complex into quercetin ITC titration in Figure 3b.

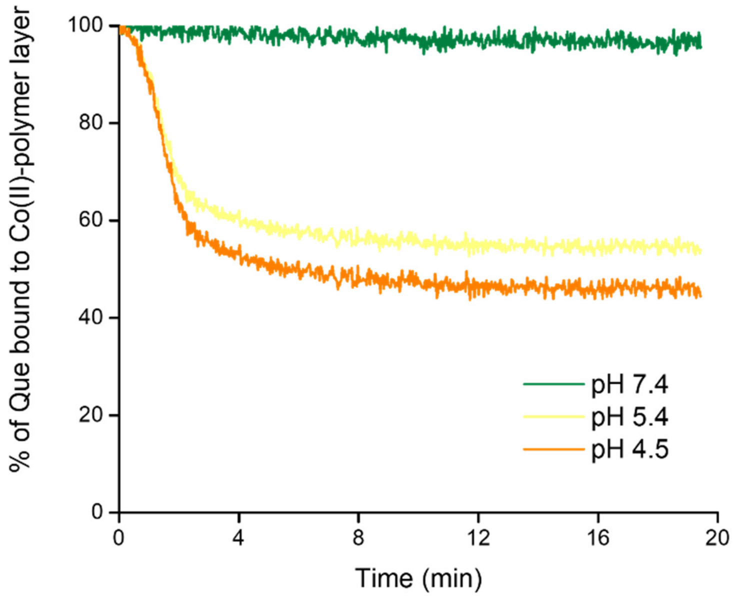

Figure S6. Release profiles of quercetin from the metal-polymer layer at different pH values. The curves of the (%) amount of Que bound are obtained by normalising the mass absorption values (i.e. the mass change curves of step 6 in Figure 5) by the maximum amount of Que bound as a function of time. The curves refer to a single QCM-D

measurement for each pH value. The amount (%) of Que released reported in Table 4 was instead calculated as the average value from at least three experiments.
